# Supplementary material for: Sodium–Glucose Cotransporter-2 Inhibitors Could Help Delay Renal Impairment in Patients with Type 2 Diabetes: A Real-World Clinical Setting
Source: J Clin Med. 2022 Sep 6;11(18):5259. doi: 10.3390/jcm11185259 (PMC9502124; doi:10.3390/jcm11185259)
Supplement: Supplementary file 1 [file jcm-11-05259-s001.zip › jcm-1864904-supplementary.pdf]

Additional file S1: Supplementary information

Table S1. Approval of sodium–glucose co-transporter-2 (SGLT2) inhibitors for glucose control in patients with type 2 diabetes by MFDS (accessed June 2021)

Drugs	Dapagliflozin	Empagliflozin	Ertugliflozin	Ipragliflozin
eGFR criterion				
(ml/min/1.73 m^2)				
Do not start†	< 60	< 60	< 60	< 60
Not recommended‡	< 45	< 45	< 60	< 60
Contraindicated	< 30	< 30	< 30	< 30

†Should not be started in patients with type 2 diabetes for the purpose of improving blood glucose control. ‡Should be discontinued if eGFR is consistently lower than the corresponding range. eGFR: estimated glomerular filtration rate

Table S2. Corresponding codes for diagnoses and treatments

Category	Codes
**Diagnoses**	**ICD-10-CM code**
Hypertension	I10, I11, I12, I13, I15
Dyslipidemia	E78
Myocardial infarction	I21, I22
Stroke	I60-I64
Heart Failure	I11.0, I13.0, I13.2, I50
Atrial fibrillation	I48
Peripheral artery disease	I70, I73
Diabetic retinopathy	E11.3, E13.3, E14.3, H28.0, H35.8, H36.0
Diabetic neuropathy	E10.4, E11.4, E13.4, E14.4, G59.0, G63.2
Diabetic nephropathy	E11.2, E13.2, E14.2, N08.3
Cancer	C00-C97
End stage renal disease	E11.22, E12.22, E13.22, E14.22
Dialysis	Z99.2
Kidney transplantation	Z94.0
**Treatments**	**ATC code**
SGLT2 inhibitors	A10BD15, A10BD20, A10BK01, A10BK03, A10BK04, A10BK05
DPP-4 inhibitors	A10BD07, A10BD08, A10BD09, A10BD10, A10BD11, A10BH, A10BH01, A10BH02, A10BH04, A10BH05, A10BH06
Metformin	A10BA02, A10BD, A10BD02, A10BD05, A10BD07, A10BD08, A10BD10, A10BD11, A10BD15, A10BD20, A10BH
Sulfonylurea	A10BB09, A10BB12, A10BD02
Thiazolidinedione	A10BD, A10BD05, A10BD09, A10BG, A10BG03
Meglitinide	A10BX02, A10BX03

α-glucosidase inhibitors	A10BD, A10BF01, A10BF03
Insulin	A10AB01, A10AB04, A10AB05, A10AB06, A10AC01, A10AD01, A10AD04, A10AD05, A10AD06, A10AE04, A10AE05, A10AE06, A10AE54
GLP-1 receptor agonists	A10AE54, A10BJ02, A10BJ05
Statins	C10AA01, C10AA04, C10AA05, C10AA07, C10AA08, C10BA02, C10BA05, C10BA06, C10BX, C10BX03, C10BX09, C10BX10, C10BX16
Ezetimibe	C10AX09, C10BA02, C10BA05, C10BA06
Fibrates	C10AB02, C10AB04, C10AB05, C10BA03
Calcium channel blockers	C07FB02, C08CA01, C08CA02, C08CA04, C08CA05, C08CA07, C08CA09, C08CA11, C08CA12, C08CA13, C08DA01, C08DB01, C09BB05, C09DB01, C09DB02, C09DB04, C09DB06, C09DB07, C09DB08, C09DB09, C09DX, C09DX03, C10BX, C10BX03, C10BX09
ACEIs	C09AA01, C09AA02, C09AA03, C09AA04, C09AA05, C09AA08, C09AA15, C09BB05
ARBs	C09CA01, C09CA02, C09CA03, C09CA04, C09CA06, C09CA08, C09CA09, C09CA10, C09DA01, C09DA02, C09DA03, C09DA04, C09DA06, C09DA07, C09DA09, C09DA10, C09DB01, C09DB02, C09DB04, C09DB06, C09DB07, C09DB08, C09DB09, C09DX, C09DX03, C09DX04, C10BX, C10BX10, C10BX16
β-blockers	C01CA02, C07AA05, C07AA12, C07AB02, C07AB03, C07AB06, C07AB07, C07AB08, C07AB09, C07AB12, C07AG, C07AG01, C07AG02, C07CB03, C07FB02
Thiazides	C03AA03, C03EA01, C09DA01, C09DA02, C09DA03, C09DA04, C09DA06, C09DA07, C09DA10, C09DX03
Aldosterone antagonists	C03DA01, C03DB01, C03EA01
Loop diuretics	C03BA11, C03CA01, C03CA04

Platelet aggregation inhibitors	B01AC, B01AC06, B01AC18, B01AC23, B01AC30
P2Y12 inhibitors	B01AC04, B01AC22, B01AC24, B01AC30
Warfarin	B01AA03
NOACs	B01AE07, B01AF01, B01AF02, B01AF03

SGLT2: sodium–glucose cotransporter-2; DPP-4: dipeptidyl peptidase-4; GLP: glucagon–like peptide; ACEI: angiotensin-converting-enzyme inhibitor; ARB: angiotensin II receptor blocker; NOAC: new oral anticoagulant

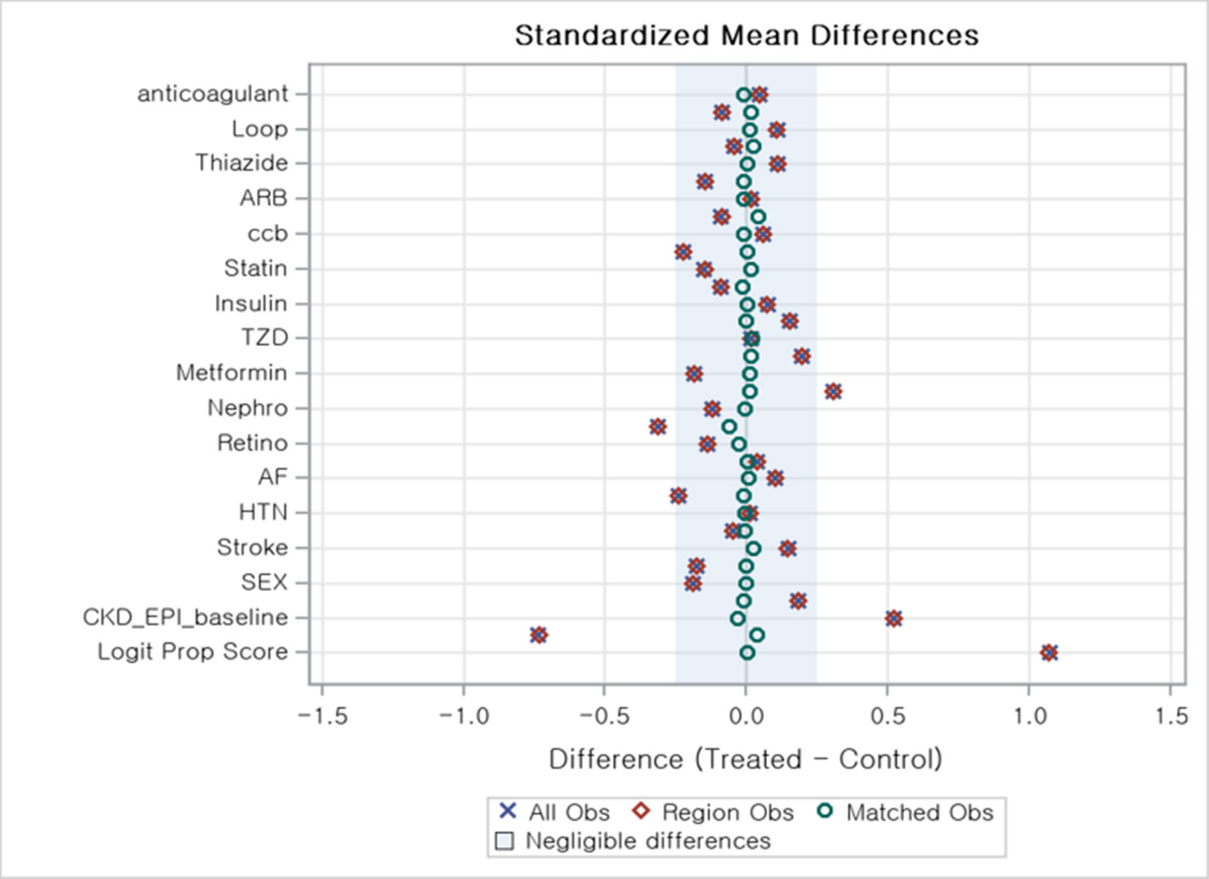

Figure S1. Standardized mean differences before and after matching

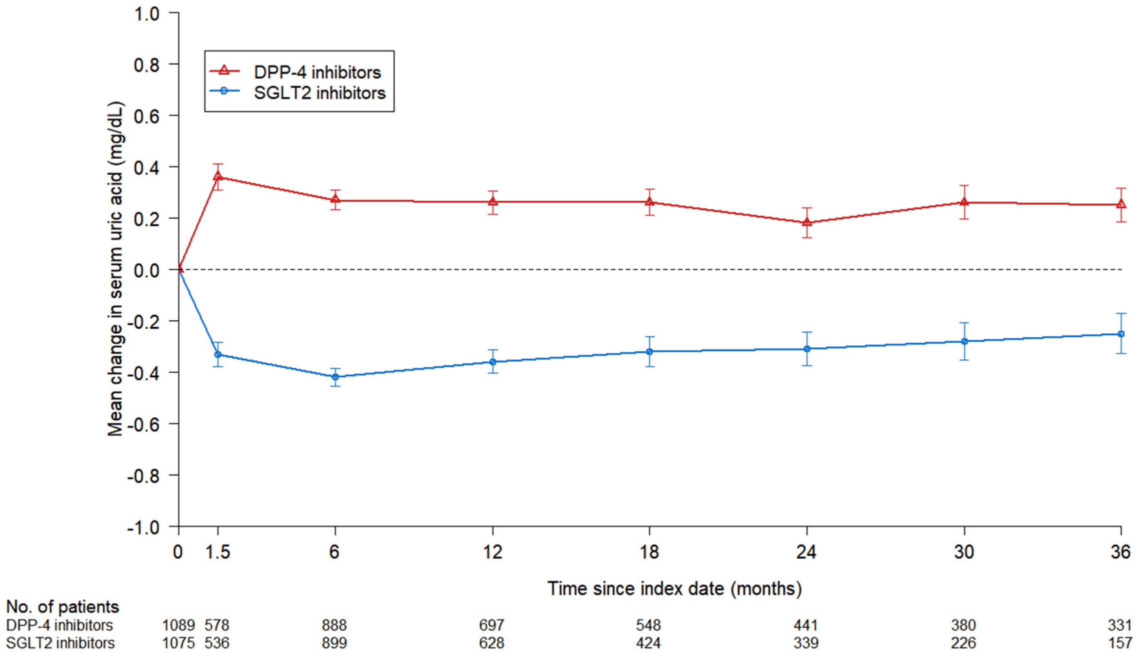

Figure S2. Change in SUA in SGLT2 inhibitors group and DPP-4 inhibitors group

Error bars indicate standard errors. Numbers below the graph refer to the number of patients at each time point. SUA: serum uric acid; SGLT2: sodium–glucose cotransporter-2; DPP-4: dipeptidyl peptidase-4.
